# Maternal vaccination as an additional approach to improve the protection of the nursling: Anti-infective properties of breast milk

**DOI:** 10.1016/j.clinsp.2022.100093

**Published:** 2022-08-10

**Authors:** Yingying Zheng, Simone Correa-Silva, Patricia Palmeira, Magda Carneiro-Sampaio

**Affiliations:** a Department of Pediatrics, Faculdade de Medicina, Universidade de São Paulo, São Paulo, SP, Brazil; b Universidade Paulista, UNIP, São Paulo, SP, Brazil; c Laboratory of Medical Investigation (LIM-36), Department of Pediatrics, Hospital das Clínicas HCFMUSP, Faculdade de Medicina, Universidade de São Paulo, São Paulo, SP, Brazil

**Keywords:** Maternal immunization, Breast milk, Bioactive factors, Secretory IgA antibodies, Anti-SARS-CoV-2 vaccines

## Abstract

Human milk constitutes a secretion with unique functions of both nourishing the nursling and providing protection against enteric and respiratory infections, mainly due to its content of secretory IgA antibodies but also due to the presence of a plethora of bioactive factors. Specific IgA antibodies are produced locally by plasma cells derived from B lymphocytes that migrate from other mucosae to the mammary gland during lactation, particularly from the gastrointestinal and respiratory tracts. Therefore, here, the authors will provide a comprehensive review of the content and functions of different nutritional and bioactive anti-infectious components from breast milk, such as oligosaccharides, lactoferrin, haptocorrin, α-lactalbumin, k-casein, lysozyme, lactoperoxidase, mucin, fatty acids, defensins, cytokines and chemokines, hormones and growth factors, complement proteins, leukocytes and nucleic acids, including microRNAs, among many others, and the induction of antibody responses in breast milk after maternal vaccination with several licensed vaccines, including the anti-SARS-CoV-2 vaccine preparations used worldwide. Currently, in the midst of the pandemic, maternal vaccination has re-emerged as a crucial source of passive immunity to the neonate through the placenta and breastfeeding, considering that maternal vaccination can induce specific antibodies if performed during pregnancy and after delivery. There have been some reports in the literature about milk IgA antibodies induced by bacterial antigens or inactivated virus vaccines, such as anti-diphtheria-tetanus-pertussis, anti-influenza viruses, anti-pneumococcal and meningococcal polysaccharide preparations. Regarding anti-SARS-CoV-2 vaccines, most studies demonstrate elevated levels of specific IgA and IgG antibodies in milk with virus-neutralizing ability after maternal vaccination, which represents an additional approach to improve the protection of the nursling during the entire breastfeeding period.

## Highlights

• Human milk is an essential source of immunonutrients for the nursing infant.
• Maternal vaccination provides specific IgA and IgG antibodies in breast milk.
• Milk from vaccinated mothers contains anti-SARS-CoV-2 antibodies that may protect the infant.

## Introduction

Knowledge about the physiological immaturity of the immune system in the first years of life, especially its effector mechanisms, is essential to understanding the characteristics of infectious diseases in this period of life. It is well known that newborns and infants are more vulnerable to serious infections by a wide variety of pathogens, such as extra- and intracellular bacteria, viruses, and fungi than older children and adults.

The immune system begins to develop very early in intrauterine life; in the first month, stem cells with the capacity to give rise to leukocyte progenitors are found in the yolk sac.^1^ While most other organs and systems are already developed at birth, the immune system has a functional immaturity that will develop and mature throughout childhood and adolescence as exposure to various antigens occurs.^2^ The full development of the cellular and humoral elements, and consequently, of the immune effector mechanisms, occurs at different periods throughout life. Taken together, the mechanisms of innate immunity develop earlier than adaptive immunity, and among the components of adaptive immunity (T and B lymphocytes), the full capacity to form antibodies is the last to be acquired, which only occurs in late childhood or early adolescence for serum IgA antibodies.^3^

Thus, the increased susceptibility of newborns and infants to infections is, to a large extent, compensated by the passive acquisition of immunity conferred by two crucial mechanisms: transplacental transmission of antibodies and breastfeeding, which provide transient systemic and mucosal protection, respectively, during this period.^4^ This knowledge is relevant to understanding the infant's immune response to different vaccine types. Immunization during pregnancy provides double protection, as it prevents infections in both the mother and fetus and in the latter, in addition to preventing intrauterine infection, maternal vaccination induces an IgG antibody response that will be transplacentally transmitted to the fetus, potentially modifying the severity of neonatal diseases.^5^

The transplacental transport of IgG antibodies from the mother to the fetus provides the neonate with temporary protection against pathogens to which the mother was exposed during her lifetime. At the same time, as the infant comes into contact with environmental and vaccine antigens, it starts to develop its own antibody repertoire, concomitantly with the drop in the antibody levels acquired from the mother. It has been suggested that the half-life of transferred maternal IgG in infants is 21–28 days.

It is assumed that this transport is restricted to IgG class antibodies and that it is done fundamentally by the binding of the Fc portions of these molecules with FcRn receptors present in syncytiotrophoblast cells of the placenta. Thus, the concentration of maternal IgG acquired by the newborn during pregnancy is influenced by the levels of total and pathogen-specific IgGs, IgG subclasses, gestational age at birth, and the coexistence of maternal infections that increase or decrease the transport of antibodies or affect the integrity of the placenta.^4^ Understanding the factors that affect the placental transmission of IgG antibodies is essential to exploring this mechanism for the benefit of the newborn. Based on this concept, maternal immunization is a strategy that allows the transfer of high levels of specific IgG antibodies to the neonate, providing systemic protection for weeks after birth, despite reports showing that high levels of postimmunization maternal IgG antibodies may mitigate the infant antibody response at the time of the first dose of active immunization.^6^

Pregnancy is a period of high risk for infection and increases in both maternal and fetal mortality relative to the general population have already been reported during the H1N1 epidemic^7^ and now with COVID-19.^8^ Thus, maternal immunization is a good strategy that can prevent such diseases in pregnant women, fetuses, and newborns, as pregnant women are a healthy population that responds well to vaccines, which may be administered during prenatal care.

Another crucial mechanism of passive protection that will be the focus of our review is the protective role of a plethora of bioactive factors, including immune cells and antibodies, by colostrum and breast milk. It is well known that the bioactive factors present in human milk will play different roles on mucosal surfaces of the infant's gastrointestinal and respiratory tracts, helping in the maturation of the epithelium, stimulating the development of the immune system and helping to establish a healthy microbiota without generating harmful inflammatory reactions.^9^

The components of adaptive immunity, such as T and B lymphocytes, reach the mammary gland through a complex homing mechanism originating from mucosal surfaces upon stimulation, mainly in the gut and airways.^10^ The immune system associated with the mucosal membranes, present in the respiratory and gastrointestinal tracts, is interconnected and bridges the gap between the external and internal environments of the human body. This system has to perform a complex task – it must remain tolerant against innocuous environmental, nutritional and microbial antigens to ensure organ function, but it must also promptly respond to invasive pathogens.^11^ The intestine-mammary gland axis is essential in the immune modulation observed in breast milk, in which it can be observed that the intestinal and breast mucosae are perfectly synchronized, establishing complementary interactions.^12^

The mammary gland has a close relationship with the intestine, enabling the production of essential nutrients for the baby's growth and development and also capable of producing compounds that meet the baby's microbiological, immunological, and neuroendocrine needs.^13^ It appears that such factors can transmigrate to the infant and play a key role in the infant's immune response and immune training during early life.^14^ In addition, short- and long-term benefits associated with breastfeeding have been reported.^12^

Numerous studies have shown that the concentrations of nutrients and bioactive compounds in human milk are highly dynamic and that their modifications aim to meet the specific needs of infants. Breastfeeding promotes the proper growth of the child but also covers the needs of bioactive compounds important for the development of the child's biological systems. Thus, it is important to understand that nutrients often participate in several compartments in addition to the child's nutritional status.^15^

## Nutritional components with metabolic and anti-infectious functions in human milk

Breast milk considered the ideal and complete food for newborns and infants, offers all the necessary nutrients for the newborn's development in the first 6-months of life. Exclusive breastfeeding is recommended by the WHO until 6 months of age, followed by the combination of breast milk and supplementary feeding until 2 years of age or older.^16^

Throughout lactation, milk undergoes a gradual change in its composition, being classified as colostrum, transitional milk, and mature milk. Colostrum, produced up to the seventh day after delivery and in small amounts compared to mature milk, is rich in proteins, bioactive components such as SIgA, lactoferrin, leukocytes, and growth factors but has low concentrations of carbohydrates and fat, suggesting that its main function is not nutritional but immunological, offering immediate protection to the newborn.^15^^,^^17^

Transitional milk, in contrast, is produced between the seventh day and the second postpartum week and has mixed characteristics of colostrum and mature milk, supporting the infant's nutritional and developmental needs for rapid growth. From the fourth postpartum week onward, breast milk can already be considered mature. Unlike colostrum, mature milk has lower protein and immune component contents but higher concentrations of carbohydrates and fats, reinforcing its nutritional role.^18^, ^19^, ^20^ Importantly, the literature reports increased levels of some bioactive components in colostrum and milk obtained from mothers of preterm infants when compared to those of full-term infants.^18^

Both macro- and micronutrients can have immunological functions and are used at all stages of the immune response, although most immunonutrients and immune components are proteins. Nearly 400 proteins are estimated in breast milk and are classified as caseins, whey proteins, which are predominant, and mucins, the latter being present in the milk fat globule membrane.^21^ Milk protein concentrations are generally not affected by maternal diet but increase with maternal body weight for height and decrease in women who produce higher milk volumes; they are also higher in milk from women who deliver preterm babies.^22^

Human Milk Oligosaccharides (HMOs) are soluble glycans mainly composed of monosaccharides and monosaccharide derivatives that resist hydrolysis by gastrointestinal enzymes, indicating that they remain intact in the small intestine. HMOs favor lactobacilli and bifidobacteria growth in the intestinal lumen of breastfed infants, and due to their homology with receptors present in epithelial cells for common pathogens present in the mucosa, they are able to bind and inhibit microorganism adhesion to the infant intestinal mucosa.^23^

Lipids are one of the most abundant nutrients in human milk and contain essential Polyunsaturated Fatty Acids (PUFAs), including Arachidonic Acid (AA), Eicosapentaenoic Acid (EPA), or Docosahexaenoic Acid (DHA),^24^ and short-chain fatty acids derived from HMOs used by intestinal bacteria.^25^ Such compounds, in addition to providing energy, participate in the maintenance of the intestinal mucosa, playing an extremely important role in the development of the infant's various organs. The potential of Long-Chain (LC)-PUFAs in modulating inflammation and the immune response has already been demonstrated by influencing the activity of natural killer cells, cytokine production, and lymphocyte proliferation.^26^ The distribution of different lipid types in the fat composition of breast milk is the most affected by maternal diet.^27^

Vitamins and minerals participate in almost all metabolic pathways in humans. In the immune system, they are involved in several stages of the immune response, such as leukocyte activation, modulation, proliferation, and differentiation, both in the innate and acquired immune systems. They are important to antimicrobial activity, the production of immunoglobulins, and the maintenance of intestinal barrier integrity and microbiota homeostasis.^28^ Additionally, they are strong antioxidants that protect tissues from oxidative damage. Maternal nutritional and intake statuses are capable of changing the vitamin levels in milk, but minerals are little affected since they are regulated by homeostatic mechanisms.^29^

## Bioactive components with immune functions

The beneficial effect of breastfeeding on the immune function of the nursling is already established; interestingly, the milk's immunological components are poorly affected by the maternal nutritional status but instead are affected by her immune status, such as the presence of fever, allergy, and mastitis, among other conditions.^30^ Recently, it has been described that infant infection is also capable of altering the immunological leukocyte and antibody composition of breast milk.^30^, ^31^, ^32^

The bioactive factors of human milk exert several functions, including antimicrobial activity, development, and modulation of immune and inflammatory responses, antioxidant activity, stimulation of intestinal growth and maturation, improvement of micronutrient absorption and availability, modulation of the microbiota, and development of the cognitive/nervous system^33^ (Table 1).

**Table 1:** Bioactive components with immunological functions in colostrum, transitional milk and mature milk from mothers of term infants.

	Component/Nutrient (Unit)	Colostrum	Transitional milk	Mature Milk	Immune action
**Antibodies**	Secretory IgA antibody (g/L)^21^	5.45	1.5	1.0	Perform “immune exclusion” (bind to bacteria, prevent microbial adhesion and penetration through the epithelium, without triggering inflammatory reactions)^9^
	IgM antibody (g/L)^34^	1.13	0.15	0.05	Perform “immune exclusion”, has a compensatory activity in IgA-deficient patients^9^
	IgG antibody (g/L)^34^	0.53	0.04	0.03	Neutralizing and opsonizing activities, complement system activation and antibody-dependent cytotoxicity^9^
**Carbohydrate**	Oligosaccharides (g/L)^35^	16	16	14	Promotes the growth of lactobacilli and bifidobacteria; due to the homology of epithelial cell surface receptors, they inhibit pathogen adhesion to the infant gut^3^
**Proteins**	Lactoferrin (g/L)^18^	5.71	4.32	3.64	Forms lactoferricin, limiting bacterial growth by removing essential iron; stimulates cytokine production; enhances mucosal immunity, Natural Killer (NK) cell activity and macrophage cytotoxicity; exerts activity against viruses and fungi^36^
	Haptocorrin (g/L)^37^	4.83±1.23		3.17±1.77	Antibacterial activity and facilitates the uptake of vitamin B12 by the infant's intestinal cells^36^
	α-Lactalbumin (g/L)^33^	4.56±0.41	3.52±0.27	2.85±0.24	Antimicrobial activity and binds to oleic acid to form a compound called HAMLET (a molecular complex that kills tumor cells by a process resembling programmed cell death)^9^
	k-Casein (mg/L)^33^	860±90	800±100	550±50	Soluble receptor analog with inhibitory activity for adhesion of *Helicobacter pylori* to the human gastric mucosa^36^
	α1-Antichymotrypsin (mg/L)^38^	670	95	12	Possible actions in the nursling's gut: inhibition of DNA polymerase; IL-2 stimulation of T cells; neutrophil superoxide production; chemotaxis^39^
	Osteopontin (mg/L)^33^	180±10		138±9	Induces a Th1 response^40^
	Lactoperoxidase (mg/L)^41^			0.77±0.38	Catalyzes the oxidation of thiocyanate from saliva, forming hypothiocyanate, which can kill both gram-positive and gram-negative pathogenic bacteria^36^
	Mucin (μg/mL)^34^			554±52	Inhibition of enteric bacteria binding to intestinal cells^9^
	Bile Salt-Stimulated Lipase (BSSL) (μg/mL)^42^	71.49±40.61		236.90±52.75	Binds to DC-SIGN receptor in dendritic cells and prevents its interaction with HIV-1, leading to the blocking of HIV-1 transfection of CD4^+^ T cells^43^
	Lactadherin (µg/mL)^44^		139	66±5	Present in the milk fat globule membrane; binds to human rotavirus, preventing viral attachment to host epithelial cells^45^
	Lysozyme (µg/mL)^18^	81.7	81.9	84.4	Hydrolyzes bacterial walls from gram-positive bacteria and inner proteoglycan matrix in the membranes of gram-negative bacteria^36^
	Soluble CD14 (sCD14) (µg/mL)^18^	40.67	30.80	21.20	Immunomodulatory activity on the immune and inflammatory responses in the infant gut^9^
	Trefoil factor 3 (pmoL/L)^46^	1540	310	80	Downregulates anti-inflammatory cytokines and promotes human β-defensin expression in intestinal epithelial cells^47^
	Osteoprotegerin (nM/L)^48^	2.8‒40.6	0.7‒39.9	2.9‒32.1	Binds to TNF-Related Apoptosis-Inducing Ligand (TRAIL) and induces caspase-dependent apoptosis, especially in Th1 cells, regulating the Th1/Th2 balance of the infant immune system^9^
**Fatty acids**	Oleic acid (Omega-9)	35% of total fat^49^		(15.07 mg/g)^25^	Stimulates the synthesis of ROS^50^
	LC-PUFA – AA (Omega-6)	26% of total fat^49^		(5.62 mg/g)^25^	Precursor of eicosanoids, promotes inflammatory processes by producing prostaglandins and leukotrienes, stimulates synthesis of ROS^50^
	LC-PUFA – EPA/DHA (Omega-3)	0.6% of total fat^49^		(0.89 mg/g)^25^	Inhibits the immune response, decreasing monocyte and neutrophil chemotaxis and the production of proinflammatory cytokines^50^
**Antimicrobial peptides**	β-Defensin 1 (ng/mL)^18^	110.9	185.4	66.9	Promotes monocyte-derived dendritic cell maturation and inhibits their apoptosis; promotes neonatal CD4 T-cell proliferation and activation^51^
	β-Defensin 2 (ng/mL)^18^	745.6	397.5	197.8	Antimicrobial activity against a range of bacteria, including common pathogens that cause infant diarrhea and gram-negative nosocomial bacteria^52^
	α-Defensin-5 (pg/mL)^18^	78.13	78.13	78.13	Protects against gastrointestinal and systemic infections and participates in the development of the mucosal immune system; possible inhibitory effect against SARS-CoV-2^53^^,^^54^
	Cathelicidin-derived antimicrobial peptide (LL37) (pg/mL)^18^	390.63	390.63	390.63	Antibacterial activity against both gram-positive and gram-negative bacteria; possible inhibitory effect against SARS-CoV-2^53^^,^^55^
**Nonproteins**	Nucleotide (µmoL/L)^56^	16.7‒292.0	16.6‒208.1	10.2‒240.0	Modulation of lymphocyte and NK-cell maturation, activation and proliferation, and immunoglobulin production; reduces enterobacteria and increases bifidobacterial counts in the fecal microbiota^9^
	miRNA (miRNA-Let-7a, miRNA-30B and miRNA-378, respectively) (Log10 2^–ΔΔCt^)^57^	2.58±0.67, 4.05±0.61 and 4.64±0.69		2.39±0.62, 4.92±0.57 and 3.62±0.77	Regulation of development of monocyte and Treg cells; differentiation and maturation of B and T cells; proliferation of granulocytes^58^

* Colostrum, produced up to the seventh day after delivery; Transitional milk, produced between the seventh day and the second postpartum week; Mature milk, produced from the fourth postpartum week onward. ROS, Reactive Oxygen Species.

A proteomic study performed with samples collected during a twelve-month lactation period revealed that 115 proteins were identified in the whey fraction of human milk and, of that, a group of 40 proteins was identified to exert immune-stimulating functions, and whose levels were higher in colostrum and one-month milk whey, which is the period of increased infant susceptibility to infection. These proteins included α-1-antitrypsin, α-lactalbumin, carbonic anhydrase 6, chordin-like protein 2, galectin-3-binding protein, lactadherin, lactoferrin, prolactin-inducible protein, and tenascin.^59^

## *Hormones and growth factors*

Several hormones have been described in human milk and are involved in the proliferation and differentiation of the newborn's immature cells. In the hypothalamic-pituitary-adrenal axis, Adrenocorticotropic Hormone (ACTH) has been described but is degraded in the gastrointestinal tract of breastfed infants.^60^ ACTH is responsible for the release of cortisol, which is absorbed, resulting in a good correlation between the nursling's serum levels and milk levels. Cortisol has immunosuppressive and anti-inflammatory effects, increases leukocyte counts, suppresses B- and T-cell activation, and inhibits prostaglandin and leukotriene production. Cortisol and its inactive form, cortisone, are also present in higher concentrations in the morning, helping to establish the circadian rhythm of the infant.^61^

The highest concentrations of melatonin are found in colostrum compared with mature milk, this hormone is also present in higher levels at night and at dawn than during the day, helping the nursling in the establishment of the circadian rhythm, and its protective antioxidant, anti-inflammatory, anti-apoptotic, and immunomodulatory effects have already been described.^62^

The adenohypophysis hormones, including Growth Hormone (GH), Thyroid-Stimulating Hormone (TSH), Follicle-Stimulating Hormone (FSH), Luteinizing Hormone (LH), and prolactin, are present in human milk. Other thyroid hormones stimulated by Thyroid-Stimulating Hormone (TSH), such as Thyroid Releasing Hormone (TRH), Triiodothyronine (T3), Thyroxin (T4) and calcitonin, are also present, as well as Parathyroid Hormone (PTH) and PTH-related peptides.^63^

Human milk also contains numerous appetite, growth, and obesity-related hormones, including leptin, Insulin-like Growth Factor-1 (IGF-1), glucose, adiponectin, insulin, obestatin, somatostatin, resistin, and ghrelin; higher concentrations of milk leptin were associated with lower BMIZ (BMI-for-age z score), and insulin was associated with lower infant weight, relative weight, and lean mass.^64^, ^65^, ^66^, ^67^

A variety of gastrointestinal hormones have been described in human milk, playing roles in gastrointestinal tract function, growth, and maturation. While bombesin, cholecystokinin, peptide YY, Vasoactive Intestinal Peptide (VIP) and neurotensin decrease over the first 6 weeks postpartum, Gastric Inhibitory Peptide (GIP) concentrations remain stable. These maternal hormones may partly compensate for the decreased gastric and protease activity and intestinal motility, which increase the infant's susceptibility to gastrointestinal infections, by active stimulation of these activities.^68^

Erythropoietin (EPO) levels increase throughout lactation and favor intestinal villous integrity via stimulation of intestinal epithelial cell proliferation and migration, in part by promoting resistance to TNF-induced apoptosis.^69^

Growth factors are present in breast milk, acting mainly in the proliferation and differentiation of infant gastrointestinal tract immature cells. Insulin-like Growth Factors 1 and 2 (IGF-1 and IGF-2), Epithelial Growth Factor (EGF), Vascular Endothelial Growth Factor (VEGF), Platelet-Derived Growth Factor (PDGF), Hepatocyte Growth Factor (HGF), Neural Growth Factor (NGF), Heparin Binding EGF-like Growth Factor (HB-EGF), Glucagon-Like Peptide-1 (GLP-1), and basic Fibroblast Growth Factor (bFGF) are among the growth factors detected in breast milk. Stem Cell Growth Factor-β (SCGF-β) has already been described in high concentrations in human colostrum and milk.^69^ In particular, colostrum provides the highest concentrations to meet increased postnatal needs, such as gut epithelium maturation, immune response, and neurocognitive development.^63^^,^^70^

## *Cytokines and chemokines*

The presence of an array of cytokines in human milk has already been well described, and they exert multiple functions in the infant's gastrointestinal tract.^71^ The most described cytokines in human milk are TGF-β (β1, β2 and β3), IL-1α, IL-1β, TNF-α, IFN-γ, IL-1, IL-2, IL-3, IL-4, IL-5, IL-6, IL-7, IL-8, IL-9, IL-10, IL-12, IL-13, IL-15, IL-16, IL-17, IL-18, TRAIL and LIF.^71^^,^^72^

It is suggested that they regulate inflammatory processes, prevent allergies and sepsis, promote hematopoiesis, contribute to a healthy gut and thymus development, and increase enterocyte maturation. It is assumed that they can act as immunostimulators or immunomodulators on phagocytic cells and on lymphocytes involved in the development of the child's specific immune response, acting in the prevention of hypersensitivity and allergies, probably due to the suppression of IgE production. A possible role is also described for breast milk leukocytes, promoting their activation, stimulating phagocytosis and antigen presentation, and inducing the growth, differentiation, and production of immunoglobulins by B cells.^85^ For instance, it has been shown that breastfeeding enhances the specific cellular response to Bacille Calmette-Guerin (BCG) vaccination.^73^

Transforming Growth Factor-β (TGF-β) controls the initiation and resolution of immune responses through the recruitment of lymphocytes, natural killer cells, dendritic cells, macrophages, mast cells, and granulocytes and their activation and survival; therefore, in addition to its activity as a growth factor, TGF-β is also considered a cytokine. The TGF-β effects in the neonatal intestinal tract include contributions to intestinal maturation and host defense, induction of IgM class switching to IgA in B lymphocytes, and induction of intestinal immunoglobulin production.^74^ TGF-β and IL-10 play important roles in the maintenance of intestinal homeostasis, inducing Foxp3+ regulatory T-cells, which in turn produce IL-10; both cytokines are involved in tolerance induction, providing an immunoregulatory milieu in antigen presentation and preventing excess activation of T-helper 1 (Th1) or Th2 pathways.^75^

Regarding colony-stimulating factors, human colostrum contains the greatest concentrations of G-CSF, followed by GM-CSF and M-CSF.^76^ Cytokine concentrations are also significantly different when measured in colostrum, transitional or mature milk from the same mother, and are almost always the highest in colostrum and the lowest in mature milk. Cytokines and growth factors in milk are generally low relative to other molecules, although TGF-β2 is the most abundant. When expressed as a percentage of the total protein in breast milk, the relative percentages of all molecules except TGF-β2, IL-10, IL-13, and TNF-α are highest in colostrum and decrease significantly during the 1^st^ postpartum month.^18^

Breast milk also contains soluble receptors or cytokine antagonists that contribute to its anti-inflammatory profile. IL-1Ra competes with IL-1α/IL-1β for receptor binding, representing a control mechanism to modulate IL-1 inflammatory effects, and is present in colostrum and milk at equivalent concentrations. The soluble forms of IL-6 (IL-6R) and TNF-α (sTNF RI and sTNF RII) receptors are present in higher concentrations in colostrum than in mature milk, and by binding, they are able to modulate their biologic effects.^77^

The chemokines MCP-1/CCL2, MIP-1α/CCL3, MIP-1β/CCL4, RANTES/CCL5, MCP-3/CCL7, eotaxin/CCL11, MIP-3/CCL20, TECK/CCL25, CTAK/CCL27, MEC/CCL28, GROα/CXCL1, IL-8/CXCL8, MIG/CXCL9, IP-10/CXCL10, SDF-1α/CXCL12a, Fractalkine/CX3CL1 and MIF (macrophage migration inhibitory factor), a Chemokine-Like Function (CLF) chemokine, have already been described in human colostrum and milk.^31^^,^^78^ In addition to their chemotactic role in leukocyte recruitment to human milk, it has already been demonstrated that some chemokines are also bactericidal to some gram-negative and gram-positive bacteria, parasites, and fungi.^79^

## *Complement system*

A proteomic study of human milk recently identified several complement cascade proteins, with those of the alternative pathway being the most abundant. The majority of proteins involved in activating the cascade were present in higher concentrations during early lactation and declined in later stages.^80^ In the infant's gut, it has been suggested that complement activation promotes bacteriolysis, enhances phagocytosis, and neutralizes viruses.^81^ However, the inhibitory proteins of the cascade, such as Clusterin (CLU), Vitronectin (VTN), Complement Decay-accelerating factor (CD55), complement factor I, and complement factor H, were as abundant as the activating proteins, probably to minimize the triggering of an inflammatory process, especially in early lactation.^80^

## *MicroRNAs*

It has been shown that microRNAs, small noncoding RNAs that regulate gene expression at the posttranscriptional level, are present in breast milk, carried by extracellular vesicles, including exosomes, which confer resistance to RNase digestion and tolerance to the low pH of the gastrointestinal tract, which could make them available to reach all the targeted organs or perform their functions locally.^82^ It has been hypothesized that these microRNAs can modulate the expression of genes involved in several physiological processes, including energy metabolism, immunological functions, and cognitive development. There is also evidence of the endogenous synthesis of breast milk miRNAs within the lactating mammary epithelium.^83^

## Leukocytes

Leukocytes, epithelial cells, and stem cells are reported to be present in human milk. Leukocytes are noteworthy, and their numbers are higher in colostrum than in mature milk, probably reflecting the fact that the tight junctions of the mammary epithelium are looser during the first days after birth. However, it is important to emphasize that the volume of mature milk consumed is significantly higher, maintaining significant cell intake by the infant. Macrophages (40%–50%) and neutrophils (40%–50%) are present in much higher numbers than lymphocytes (5%–10%), which mostly include T-lymphocytes (∼80%), while only 5% to 6% are B cells.^84^ These cell types are present in an activated phenotype, and their presence in milk is due to transepithelial migration into mammary tissue stimulated by specific chemokines in the mammary gland.^31^^,^^85^ Interestingly, other types of leukocytes (basophils, eosinophils, mast cells) and platelets, which are usually related to inflammation, are rarely found.^19^ It is worth mentioning that viable leukocytes from milk have been isolated in feces from infants fed human milk,^85^ suggesting that these cells could remain intact in the gut. It has been shown that some of these cells are able to cross epithelial barriers in animal models and are engrafted in the offspring's tissues and organs.^86^^,^^87^

## Microbiota

The human milk microbiome or microbiota is now recognized as harboring many bacteria, viruses, fungi, and yeasts, which are continuous sources of commensal and potentially probiotic bacteria for the infant's gut.^88^ The composition of the human milk microbiome is influenced by several factors, including maternal (mode of delivery, diet and BMI, diseases, perinatal antibiotics, and postnatal psychosocial distress), neonatal (gestational age, sex, and birth weight), and environmental factors (geographical study location and seasonality, lifestyle, social network and collection methods) and factors related to human milk itself (lactation period, pasteurization and levels of oligosaccharides, milk fatty acids, hormones, immune cells, and antibodies).^89^

More than 207 bacterial genera have been identified in human mature milk, recognized as one of the main sources of bacteria for the infant's intestine, and are composed mainly of the *Firmicutes* and *Proteobacteria* phyla, with 12 genera prevailing in milk from most lactating mothers: *Pseudomonas, Staphylococcus, Streptococcus, Elizabethkingia, Variovorax, Bifidobacterium, Flavobacterium, Lactobacillus, Stenotrophomonas, Brevundimonas, Chryseobacterium,* and *Enterobacter*.^90^ It is worth mentioning that the composition of the fecal microbiota of the nursling reflects that found in the breast milk, and the genera *Lactobacillus, Staphylococcus, Enterococcus,* and *Bifidobacterium* are frequently shared between breast milk and infant feces, as described in several studies.^90^, ^91^, ^92^

It has been suggested that the microbiota is composed of bacteria present in the nasopharynx and oral cavity of the nursling or the nipple skin. However, analysis with precolostrum, that is, milk without contact with the newborn, verified the presence of bacteria in milk.^93^ Another hypothesis for the origin of the bacteria present in breast milk is that nonpathogenic bacteria are taken up by dendritic cells in the gut lumen and are carried to the mammary glands by the intramammary axis.^94^ Corroborating this, the presence of live bacteria attached to the extracellular matrix of immune cells has been reported.^95^ In addition, a study performed with pregnant and lactating mice showed that bacteria can translocate from the gut to mammary glands.^96^

These human milk bacteria can play several roles in the infant's intestinal mucosa, contributing to reductions in infection incidence and severity by different mechanisms, including prevention of pathogenic bacteria adhesion and colonization by competitive exclusion; production of antimicrobial compounds; improvement of the intestinal barrier by increasing mucin production and reducing intestinal permeability; enhancement of nutrient metabolism and absorption; and stimulation of the gut-brain axis. They are also essential for the production and supply of vitamins K and B12 and short-chain fatty acids to the infant.^97^, ^98^, ^99^

## Antibodies from human milk

Antibodies from human milk reflect antigen-specific Mucosa-Associated Lymphoid Tissue (MALT) stimulation in both the intestine and the airways. Human colostrum and milk contain all immunoglobulin isotypes, with Immunoglobulin A (IgA) present in the highest concentrations, totaling 80% to 90% of the total immunoglobulins in human milk, followed by IgM and IgG, the latter being present at low concentrations. SIgA in breast milk has been well proven to exhibit specificity for a variety of common intestinal and respiratory microorganisms.^100^

IgA is the most abundant class of antibodies in the human adult organism (with higher concentrations in mucous secretions and the second most abundant immunoglobulin in the blood); ∼40 mg/kg body weight of polymeric IgA is translocated to the intestinal secretions per day, more than the total daily production of IgG.^101^ IgA can be found in monomeric, dimeric, or trimeric forms, with the monomeric form being predominant in serum and the polymeric form being predominant in external secretions, where it is called Secretory IgA (SIgA). SIgA contains the J (Joining) chain, which binds 2 or 3 IgA molecules, and the Secretory Component (SC), which protects the molecular complex “SIgA” against the action of proteases abundant in the digestive tract.^10^

IgA molecules and the J chain are produced by plasmocytes present in the lamina propria of the mucosa, while the SC is derived from the Polymeric Immunoglobulin Receptor (pIgR), a transmembrane glycoprotein present in epithelial cells. This pIgR is responsible for the transcytosis of IgA across the epithelium to the luminal side, where the receptor is cleaved and the extracellular portion, which is approximately 80 kDa in size, remains associated with the molecular IgA complex, constituting the SC of the SIgA molecule. A significant amount of free SC is also released in mature human milk (783 μg/mL),^102^ and its highly glycosylated nature has been demonstrated to show *in vitro* protection against *Clostridium difficile* toxin A, *Escherichia coli,* and gram-positive commensals.^103^

SIgA is found in all external secretions and in mucous membranes, with colostrum and milk having the highest concentrations among them.^10^ SIgA and SIgM are present in higher concentrations in the first few days of lactation, with SIgA reaching concentrations between 10 and 20 g/L, 4 to 5 times greater than the concentrations of IgM, 20 to 30 times greater than the concentrations of IgG, and 5 to 6 times greater than the concentrations of serum IgA.^9^^,^^104^ Sharp decreases in IgA and IgM levels are seen in milk throughout lactation, but these decreases are more than balanced by an increase in milk intake as lactation progresses, whereas IgG levels do not show any significant change when early and late lactation periods are compared.^84^

SIgA antibodies in human milk are reactive with numerous pathogens that the mother has been challenged with throughout her life, thus representing her immunological memory acquired over the years. The SIgA present in milk does not come from the blood but is produced locally by plasma cells derived from B lymphocytes that migrate from other mucosae to the mammary gland during the lactation phase, especially from the intestine and respiratory tracts. Thus, the levels of antibodies reactive with antigens from microorganisms that penetrate through the mucosae are generally higher in colostrum than in serum.^9^

Several clinical and epidemiological studies have shown that milk SIgA antibodies efficiently prevent the entry of microorganisms into tissues and represent an important protective factor for infants against infectious diarrhea and upper respiratory infections, including otitis media.^9^^,^^105^ The main function of SIgA is to act as the first line of defense against foreign antigens, binding to bacteria – both commensal and pathogenic – toxins, viruses, protozoa, and other antigenic materials such as Lipopolysaccharides (LPS), thus preventing microbial adhesion and its penetration through the epithelium without triggering inflammatory reactions that could be harmful to the newborn's mucosa in a neutralization mechanism called “immune exclusion”.^10^

It is important to mention that, although there is some digestion of all immunoglobulin classes in the gastric tract of the nursling, the stability of human milk immunoglobulins upon gastric digestion has been shown to be higher in the preterm infant than in term infants, probably due to the higher gastric pepsin activity and proteolysis in term neonates.^106^ Alternatively, it was also demonstrated that SIgA is able to remain intact and biologically active in the feces of breastfed newborns and young infants.^107^ In addition, studies assessing the digestibility of IgG in ileal aspirates and stools from infants, children, and adults have demonstrated that orally-administered IgG, from human or bovine serum as well as from bovine colostrum and milk, but not from human milk, survived gastric exposure and resisted to proteolysis in the stomach and intestinal tract, but the recovered IgG concentrations varied from study to study.^108^

## Human milk as a passive immunization against pathogens

While the placental transfer of IgG antibodies after vaccination during pregnancy is well documented, few studies exist on the induction of vaccine-specific antibodies, particularly IgA antibodies, in breast milk.^109^ It is noteworthy that transplacentally acquired systemic IgG has a short half-life,^3^^,^^110^^,^^111^ while IgA acquired by breastfeeding will be present in the infant's mucosa throughout the breastfeeding period. There have been few studies about specific IgA and IgG levels in breast milk induced by immunization and how they confer protection to the nursling. Most existing studies have only analyzed specific IgA levels, certainly because IgA is the most abundant antibody in human milk. Here, the authors present the main findings in the literature on specific IgA and IgG levels in breast milk after the administration of licensed vaccines (Table 2).

**Table 2:** Available data on milk IgA and IgG response to vaccine antigens administered during pregnancy.

Author, year and country	Vaccine type	Pathogen analyzed/ Disease	Specific IgA^a^	**Specific IgG**^a^
Shahid et al. (2002) Bangladesh^112^	Meningococcal vaccine ‒ polysaccharide from groups A, C, Y and W-135 (Menomune®, Sanofi Pasteur Inc., Swiftwater, PA)	*Neisseria meningitidis/*Sepsis and meningitis	↑	NE
Shahid et al. (1995) Bangladesh^113^	23-valent pneumococcal polysaccharide vaccine (Pnu-Immune 23®, Lederle-Praxis Biologicals, Pearl River, NY)	Pneumococcus serotypes 6B and 19F/Pneumonia, meningitis, otitis media, sinusitis, and sepsis	↑	NE
Munoz et al. (2001) USA^114^	23-valent pneumococcal vaccine (Pneumovax23®; Merck and Co. Inc., West Point, PA)	Pneumococcus serotypes 6B, 14, 19F and 23F	↑	↑
Lehmann et al. (2003) Papua New Guinean^115^	23-valent pneumococcal polysaccharide vaccine (Pneumovax II; Pasteur Mérieux, Lyon, France)	Pneumococcus serotypes 5, 7F, 14 and 23F	↑	NE
Obaro et al. (2004) Gambian^116^	23-valent pneumococcal polysaccharide vaccine (Pneumovax II; Pasteur Mérieux, Lyon, France)	Pneumococcus serotypes 4, 6B, 14, 19F and 23F	↑ SIgA	NE
Deubzer et al. (2004) Gambian^117^	Polyvalent pneumococcal polysaccharide vaccine (Pneumovax II; Pasteur Mérieux, Lyon, France)	Pneumococcus serotypes 6B and 14	↑ SIgA	NE
			↑ avidity and inhibition of pneumococcus adhesion to epithelial cells	
Schlaudecker et al. (2013) Bangladesh^118^	Inactivated influenza virus vaccine (Fluarix; GSK Biologicals, Rixensart, Belgium)	Influenza A/New Caledonia (H1N1), A/Fujian (H3N2), and B/Hong Kong/ Flu	↑ IgA and virus neutralization	NE
Abu Raya et al. (2014) Israel^119^	Tdap (Boostrix; GSK Biologicals, Rixensart, Belgium)	Pertussis toxin, filamentous hemagglutinin, and pertactin/ Whooping cough	↑	↑
Lima et al. (2019) Brazil^120^	Tdap vaccine (Boostrix; GSK Biologicals, Rixensart, Belgium)	Pertussis toxin and whole cell *B. pertussis*	=	NE
Orije et al. (2021) Belgium^121^	Pertussis-containing vaccine (Tdap, Boostrix, GSK Biologicals)	Pertussis toxin	=	↑

a Comparison of specific IgA/SIgA or IgG levels in breast milk between vaccinated and non-vaccinated mothers. (↑) Higher level was found in breast milk from vaccinated women compared to non-vaccinated ones. (=) No differences were found between vaccinated and non-vaccinated women. NE, Not Evaluated.

Vaccination both during pregnancy and during lactation may induce increases in specific antibodies in breast milk, although most studies reported vaccine administration during pregnancy.^112^, ^113^, ^114^, ^115^, ^116^, ^117^, ^118^, ^119^, ^120^, ^121^ The follow-up time for each study varied; while some analyzed antibody levels until 12 postpartum weeks,^119^, ^120^, ^121^ others followed up to 6 months or even one postpartum year.^111^, ^112^, ^113^, ^114^^,^^116^^,^^118^

As observed for total IgA and IgG, the same trend is seen for specific IgA and IgG antibodies, with higher levels in colostrum, which decrease as the milk matures. However, how long those specific antibodies remain in milk varies according to the vaccine type. It has been reported that meningococcal vaccine, 23-valent pneumococcal polysaccharide vaccine, and inactivated influenza virus vaccine can induce high specific IgA levels up to 6 months postpartum and high specific IgG levels until the 2^nd^ post-delivery month, and these antibody levels could be 4 to 5 times higher than in milk from nonvaccinated mothers.^112^, ^113^, ^114^^,^^116^, ^117^ It has also been described that the Tdap vaccine induces high levels of anti-pertussis IgA in milk, but only until the second postpartum week in one study^121^ and the eighth postpartum week in another one, but in this latter , there was a sharp drop in specific antibody levels after the 2^nd^ postpartum week.^119^

Regarding antiviral antibodies in human milk, it is worth mentioning that live-attenuated viral vaccines are contraindicated for pregnant women due to possible transplacental transmission of the attenuated virus to the fetus or into breast milk. In contrast, immunization with nonviable inactivated virus vaccines has not shown risks to the fetus and is even recommended during pregnancy.^5^ This strategy has the potential to protect the pregnant woman and fetus from serious illnesses during pregnancy, such as respiratory infections. In addition, this strategy offers protection to newborns and infants during the first months of life.^109^

Brady et al.^122^ observed that inactivated influenza vaccine induced higher specific IgA levels in breast milk than the live-attenuated vaccine; in addition, the latter was equally as safe as the inactivated vaccine, as no viable influenza viruses were detected in milk. In another study, it was also shown that colostrum from vaccinated mothers presented measles-specific IgA at five times above the protective level, but the level fell below the positive cutoff after one postpartum month.^123^

Studies on the safety of other live virus vaccines, such as varicella^124^ and measles,^125^ also did not observe the presence of those viable viruses in milk. Rubella vaccine virus^126^, ^127^, ^128^ may be excreted into human milk but may not cause clinical disease in infants. It is assumed that if infection with a live vaccine occurs, it will be well tolerated because the vaccine virus is attenuated.^129^ In contrast, yellow fever vaccination is not recommended to nursing mothers because meningoencephalitis has been reported among breastfed infants.^130^^,^^131^ Unfortunately, those studies did not evaluate the antibody response in breast milk.

Regarding oral vaccines, rotavirus vaccines are not indicated for women of childbearing age because transplacental IgG antibodies and immune components present in breast milk can reduce the infant's immune response to the administered rotavirus vaccine, but there is controversy regarding the measures adopted to minimize the neutralizing effect of breast milk consumed before and after the infant receives the rotavirus vaccine.^132^

## Human milk as a passive immunization against COVID-19

In 2020, SARS-CoV-2 infection spread globally and became pandemic. Initially, it was found that the most affected population was adults and the elderly. However, the involvement of pregnant and lactating women has also been observed.^133^ Concomitantly, the question arose as to whether maintaining breastfeeding would be a safe practice for the infant. After many questions, a consensus was reached that breastfeeding was indicated and should be the main guideline for health professionals because, in addition to offering the various benefits inherent in breast milk, studies have proven the presence of specific antibodies against SARS-CoV-2.^134^

The Centers for Disease Control and Prevention (CDC), the World Health Organization (WHO), the American College of Obstetricians and Gynecologists (ACOG), and the Royal College of Obstetricians and Gynecologists of Great Britain (RCOG), among others, indicate that pregnant women are at increased risk for severe COVID-19. Published data indicate an increased risk of hospitalization in intensive care units, the need for mechanical ventilation, and the likelihood of death in pregnant women with COVID-19 compared to nonpregnant women of childbearing age.^133^^,^^135^^,^^136^ There is also evidence of increased incidence rates of preterm birth and C-section delivery, myocardial injury, pre-eclampsia, and perinatal mortality in pregnant women with COVID-19.^137^ It was reported that pregnancy ended in preterm delivery in approximately 75% of cases of COVID-19, with 255 patients included in the study.^138^

Studies on the presence of anti-SARS-CoV-2 antibodies in milk from women infected in late pregnancy and in lactation have detected IgA, IgM, and IgG antibodies reactive with SARS-CoV-2.^139^, ^140^, ^141^, ^142^, ^143^, ^144^ While IgA is predominant in milk after natural SARS-CoV-2 infection,^145^ IgG is predominant after vaccination, reflecting differences in antibody profile programming across mucosa acquired during natural SARS-CoV-2 infection vs. intramuscular vaccination.^146^ These studies demonstrate the importance of maintaining breastfeeding at such a special time when antibodies produced in human milk can protect infants against COVID-19. Whether breast milk IgG or IgA will be more critical for neonatal protection remains unclear. In our studies, as well as in the study by Hahn-Zoric et al.,^147^ it was shown that in nursing mothers with Common Variable Immunodeficiency (CVID) in IVIG therapy or with selective IgA deficiency, increased IgG and IgM antibody levels in milk constitute a compensatory mechanism for the lack of SIgA in the mucosa, and the biological action of these antibodies was as efficient as that observed in healthy women.^147^, ^148^, ^149^

Robust SIgA responses have been reported in breast milk to SARS-CoV-2.^150^ More recently, studies have demonstrated the presence of neutralizing antibodies in breast milk after vaccination with the mRNA vaccine.^151^, ^152^, ^153^

Several studies have been published showing the presence of anti-SARS-CoV-2 antibodies in human milk after maternal immunization. Table 3 shows all the currently available studies evaluating anti-SARS-CoV-2 IgA and IgG antibodies from human milk after maternal vaccinations performed in several countries and with different technologies. This fact favors the comparative study on the vaccine response present in the mucous membranes and in breast milk. It is very interesting to emphasize that the same results were observed with different vaccine development methodologies, i.e., regardless of the vaccine used, good antibody response was achieved in breast milk. Our group had the opportunity to demonstrate that after vaccination with Coronavac (Sinovac Biotech Ltd., China and Instituto Butantan, Brazil), an inactivated SARS-CoV-2 vaccine, lactating women showed high levels of anti-protein S IgA antibodies.^154^

**Table 3:** Available data on anti-SARS-CoV-2 IgA and IgG antibodies from human milk after lactating women vaccination.

Authors/Year (Country)	Vaccine type	Sample	Specific IgA	Specific IgG
Calil et al. (2021) (Brazil)^154^	CoronaVac (Sinovac Biotech Ltd., China and Butantan Institute, Brazil)	(n = 20) Milk samples collected before and at eight additional timepoints after	↑ (anti-S1 SARS-CoV-2 IgA)	NE
Valcarce et al. (2021) (USA)^155^	BNT162b2 (BioNTech/Pfizer)	(n = 21) Plasma and milk samples collected before and at two timepoints after	↑ (anti-SARS-CoV-2 IgA)	↑ (anti-SARS-CoV-2 IgG)
	mRNA-1273 (Moderna/NIH)			
Lechosa-Muñiz et al. (2021) (Spain)^156^	1. mRNA vaccine (Moderna)	(n = 110) Blood and milk samples collected after the second vaccine dose	1 - ↑	1 - ↑
	2. ChAdOx1-S (Astrazeneca)		2 - Slightly increased	2 - Slightly increased
	3. BNT162b2 (Pfizer-BioNTech)		3 - ↑ (Anti-S1 IgA)	3 - ↑ (Anti-S1 IgG)
Juncker et al. (2021) (Netherlands)^157^	BNT162b2 (BioNTech/Pfizer)	(n = 26) Milk samples collected before and at seven timepoints after	↑ (Anti-S1 IgA)	↑ (Anti-S1 IgG)
Baird et al. (2021) (USA)^158^	BNT162b2 (BioNTech/Pfizer)	(n = 7) Milk samples collected before and at eleven additional timepoints after	↑ (Anti-S1 IgA and Anti-RBD IgA)	↑ (Anti-S1 IgG and Anti-RBD IgG)
	mRNA-1273 (Moderna/NIH)			
Selma-Royo et al. (2021) (Spain)^159^	1. BNT162b2 (BioNTech/Pfizer) and mRNA-1273 (Moderna/NIH)	(n = 75) Milk samples collected before and at six timepoints after	↑ (Anti-RBD IgA)	↑ (Anti-RBD IgG)
	2. ChAdOx1 nCoV-19 (Oxford/AstraZeneca)			
Perl et al. (2020) (Israel)^160^	BNT162b2 (Pfizer-BioNTech)	(n = 84) Milk samples collected before and at six timepoints after	↑ (Anti–S1 IgA)	↑ (Anti–S1 IgG)
Gray et al. (2021) (USA)^146^	1. BNT162b2 (Pfizer/BioNTech)	(n = 31) Blood and milk samples collected before and at two timepoints after	↑ (Anti-S1 IgA and Anti-RBD IgA)	↑ (Anti-S1 IgG and Anti-RBD IgG)
	2. mRNA-1273 (Moderna/NIH)			
Sadiq et al. (2021) (Pakistan)^161^	Sinovac or Sino Pharm (Sinovac Biotech Ltd., China)	(n = 180) Blood and milk samples collected before the 1^st^ and after the 2^nd^ dose	↑ (Anti-S1 IgA)	NE
Low et al. (2021) (Singapore)^162^	BNT162b2 (Pfizer/BioNtech)	(n = 14) Milk samples collected before and at four timepoints after	↑ (Anti-S1 IgA and Anti-RBD IgA)	↑ (Anti-S1 IgG and Anti-RBD IgG)
Narayanaswamy et al. (2022) (USA)^151^	BNT162b2 (BioNTech/Pfizer)	(n = 30) Milk samples collected at 2 timepoints before 1^st^ vaccine dose and at 11‒15 timepoints over 42‒48 days	↑ (Anti-RBD IgA)	↑ (Anti-RBD IgG)
	mRNA-1273 (Moderna/NIH)		Anti-S and anti-RBD IgA neutralizing antibodies	Anti-RBD IgG neutralizing antibodies
Yeo et al. (2022) (Singapore)^152^	BNT162b2 vaccine (Pfizer/BioNTech)	(n = 35) Milk samples across 21 days after the second dose	↑ (Anti-RBD IgA and IgM)	↑ (Anti-RBD IgG1)
			Neutralizing antibodies	Neutralizing antibodies
Perez et al. (2022) (USA)^153^	1. BNT162b2 (Pfizer/BioNTech)	(n = 30) Milk samples across 1.3 and 6 months after the second dose	↑ (Anti-RBD IgA)	↑ (Anti-RBD IgG1)
	2. mRNA-1273 (Moderna/NIH)		Neutralizing antibodies	Neutralizing antibodies with greater activity than IgA

^a^ Comparison of specific IgA/SIgA or IgG levels in breast milk between vaccinated and non-vaccinated mothers. (↑) Higher levels were found in breast milk from vaccinated women compared to non-vaccinated controls. (=) No differences were found in milk between vaccinated and non-vaccinated women. NE, Not Evaluated.

In one study, it was shown that the levels of IgA transferred through breast milk did not increase with boosting, but IgG transfer increased significantly after the boost, resulting in high levels of IgG delivered to the neonate through breast milk.^146^ Although such data have been shown, most of the works cited in this review showed increased levels of all isotypes of antibodies, mainly IgA and IgG, after booster vaccination.

Importantly, emerging data point to a critical role for breast milk IgG in neonatal immunity against several other potentially vaccine-preventable viral pathogens, including HIV, respiratory syncytial virus, and influenza.^163^^,^^164^ It is important to emphasize that IgG is present in human milk, although at lower concentrations than IgA, and increases in the IgG class are observed both in mothers who were infected and in response to maternal vaccination. The exact mechanism responsible for the influx of IgG into breast milk and the role of this antibody in protecting the infant's mucosa is still unknown, but it is probably transferred directly from the mother's blood. It was reported that milk IgG from healthy mothers has nucleotide‐hydrolyzing activity, suggesting that these antibodies neutralize bacterial and viral DNA and RNA by forming immune complexes.^165^ A study carried out in Kigali, Rwanda, with HIV-seropositive mothers identified the presence of HIV-specific IgG in 18-month milk in almost 100% of the samples, while only 40% had specific SIgA. Indeed, anti-HIV-1 IgG, probably originating from the systemic compartment, as well as SIgM, was more frequently detected than specific SIgA.^166^ This information makes us reflect on the real role of IgG in milk and demonstrates its possible importance in protecting infants.

## Conclusions

Breastfeeding represents an ingenious immunological integration between a mother and infant that has evolved over millennia to nourish and protect infants from infectious diseases during this critical period of immune vulnerability. Notably, the contents of breast milk change over time; in the early stages of lactation, SIgA antibodies, anti-inflammatory factors, and immunologically active cells provided by breast milk support the neonatal immune system. In particular, antibodies in breast milk are directed against infectious and potentially deleterious antigens that are present in the maternal environment, which is those likely to be encountered by the infant. Alternatively, vaccination during pregnancy or during lactation may induce increases in specific antibodies in breast milk, representing an additional approach to improve the protection of the nursling throughout the breastfeeding period. This infant protection strategy has been poorly explored before, and basically for respiratory diseases, but during the pandemic, it became more evident, opening precedents to be explored for other incident diseases in childhood.

## Authors’ contributions

All authors contributed to the article, writing, reviewing, editing, and approving the submitted version.

## Conflicts of interest

The authors declare no conflicts of interest.

## References

[1] Hossain Z, Reza AHMM, Qasem WA, Friel JK, Omri A. (2022). Development of the immune system in the human embryo. Pediatr Res.

[2] Ygberg S, Nilsson A (2012). The developing immune system - from foetus to toddler. Acta Paediatr.

[3] Hong DK, Lewis DB, Wilson CB, Nizet V, Maldonado YA, Remington JS, Klein JO (2016). Remington and Klein's infectious diseases of the fetus and newborn infant.

[4] Palmeira P, Quinello C, Silveira-Lessa AL, Zago CA, Carneiro-Sampaio M (2012). IgG placental transfer in healthy and pathological pregnancies. Clin Dev Immunol.

[5] Healy CM. (2012). Vaccines in pregnant women and research initiatives. Clin Obstet Gynecol.

[6] Jones C, Pollock L, Barnett SM, Battersby A, Kampmann B. (2014). The relationship between concentration of specific antibody at birth and subsequent response to primary immunization. Vaccine.

[7] Siston AM, Rasmussen SA, Honein MA, Fry AM, Seib K, Callaghan WM (2010). Pandemic 2009 influenza A(H1N1) virus illness among pregnant women in the United States. JAMA.

[8] Pierce-Williams RAM, Burd J, Felder L, Khoury R, Bernstein PS, Avila K (2020). Clinical course of severe and critical coronavirus disease 2019 in hospitalized pregnancies: a United States cohort study. Am J Obstet Gynecol MFM.

[9] Palmeira P, Carneiro-Sampaio M. (2016). Immunology of breast milk. Rev Assoc Med Bras.

[10] Brandtzaeg P. (2010). The mucosal immune system and its integration with the mammary glands. J Pediatr.

[11] Torow N, Marsland BJ, Hornef MW, Gollwitzer ES. (2017). Neonatal mucosal immunology. Mucosal Immunol..

[12] Rodríguez JM, Fernández L, Verhasselt V. (2021). The gut‒breast axis: programming health for life. Nutrients.

[13] Chu DM, Meyer KM, Prince AL, Aagaard KM. (2016). Impact of maternal nutrition in pregnancy and lactation on offspring gut microbial composition and function. Gut Microbes.

[14] Cabinian A, Sinsimer D, Tang M, Zumba O, Mehta H, Toma A (2016). Transfer of maternal immune cells by breastfeeding: maternal cytotoxic t lymphocytes present in breast milk localize in the peyer's patches of the nursed infant. PLoS One.

[15] Ballard O, Morrow AL. (2013). Human milk composition: nutrients and bioactive factors. Pediatr Clin North Am.

[16] World Health Organization (2011). http://www.who.int/topics/breastfeeding/en/.

[17] Castellote C, Casillas R, Ramírez-Santana C, Pérez-Cano FJ, Castell M, Moretones MG (2011). Premature delivery influences the immunological composition of colostrum and transitional and mature human milk. J Nutr.

[18] Trend S, Strunk T, Lloyd ML, Kok CH, Metcalfe J, Geddes DT (2016). Levels of innate immune factors in preterm and term mothers' breast milk during the 1st month postpartum. Br J Nutr.

[19] Trend S, de Jong E, Lloyd ML, Kok CH, Richmond P, Doherty DA (2015). Leukocyte populations in human preterm and term breast milk identified by multicolour flow cytometry. PLoS One.

[20] Kulski JK, Hartmann PE. (1981). Changes in human milk composition during the initiation of lactation. Aust J Exp Biol Med Sci.

[21] Lönnerdal B, Erdmann P, Thakkar SK, Sauser J, Destaillats F. (2017). Longitudinal evolution of true protein, amino acids and bioactive proteins in breast milk: a developmental perspective. J Nutr Biochem.

[22] Bauer J, Gerss J. (2011). Longitudinal analysis of macronutrients and minerals in human milk produced by mothers of preterm infants. Clin Nutr.

[23] Plaza-Díaz J, Fontana L, Gil A. (2018). Human milk oligosaccharides and immune system development. Nutrients.

[24] Fu Y, Liu X, Zhou B, Jiang AC, Chai L. (2016). An updated review of worldwide levels of docosahexaenoic and arachidonic acid in human breast milk by region. Public Health Nutr.

[25] Dai X, Yuan T, Zhang X, Zhou Q, Bi H, Yu R (2020). Short-chain fatty acid (SCFA) and medium-chain fatty acid (MCFA) concentrations in human milk consumed by infants born at different gestational ages and the variations in concentration during lactation stages. Food Funct.

[26] Ganapathy S. (2009 Sep). Long chain polyunsaturated fatty acids and immunity in infants. Indian Pediatr.

[27] Butts CA, Hedderley DI, Herath TD, Paturi G, Glyn-Jones S, Wiens F (2018). Human milk composition and dietary intakes of breastfeeding women of different ethnicity from the Manawatu-Wanganui region of New Zealand. Nutrients.

[28] Gombart AF, Pierre A, Maggini S (2020). A review of micronutrients and the immune system-working in harmony to reduce the risk of infection. Nutrients.

[29] Bravi F, Wiens F, Decarli A, Dal Pont A, Agostoni C, Ferraroni M. (2016). Impact of maternal nutrition on breast-milk composition: a systematic review. Am J Clin Nutr.

[30] Hassiotou F, Hepworth AR, Metzger P, Tat Lai C, Trengove N, Hartmann PE (2013). Maternal and infant infections stimulate a rapid leukocyte response in breastmilk. Clin Transl Immunology.

[31] Zheng Y, Corrêa-Silva S, de Souza EC, Maria Rodrigues R, da Fonseca FAM, Gilio AE (2020). Macrophage profile and homing into breast milk in response to ongoing respiratory infections in the nursing infant. Cytokine.

[32] Riskin A, Almog M, Peri R, Halasz K, Srugo I, Kessel A. (2012). Changes in immunomodulatory constituents of human milk in response to active infection in the nursing infant. Pediatr Res.

[33] Donovan SM. (2019). Human milk proteins: composition and physiological significance. Nestle Nutr Inst Workshop Ser.

[34] Mickleson KN, Moriarty KM. (1982). Immunoglobulin levels in human colostrum and milk. J Pediatr Gastroenterol Nutr.

[35] Gidrewicz DA, Fenton TR. (2014). A systematic review and meta-analysis of the nutrient content of preterm and term breast milk. BMC Pediatr.

[36] Lönnerdal B. (2003). Nutritional and physiologic significance of human milk proteins. Am J Clin Nutr.

[37] Aoki Y, Kobayashi K, Kajii T. (1992). Enzyme-linked immunoassay of haptocorrin: analysis of milk and granulocytes. Biochem Med Metab Biol.

[38] Lindberg T, Ohlsson K, Weström B. (1982). Protease inhibitors and their relation to protease activity in human milk. Pediatr Res.

[39] Urueña C, Tellería JJ, Blanco-Quirós A, Arranz E, Gomez-Carrasco JA. (1998). Alpha-1 antichymotrypsin levels are actively increased in normal colostrum. J Pediatr Gastroenterol Nutr.

[40] Lönnerdal B. (2017). Bioactive Proteins in Human Milk-Potential Benefits for Preterm Infants. Clin Perinatol.

[41] Shin K, Hayasawa H, Lönnerdal B. (2001). Purification and quantification of lactoperoxidase in human milk with use of immunoadsorbents with antibodies against recombinant human lactoperoxidase. Am J Clin Nutr.

[42] Sha L, Zhou S, Xi Y, Li R, Li X (2019). The level of bile salt-stimulated lipase in the milk of Chinese women and its association with maternal BMI. J Biomed Res.

[43] Naarding MA, Dirac AM, Ludwig IS, Speijer D, Lindquist S, Vestman EL (2006). Bile salt-stimulated lipase from human milk binds DC-SIGN and inhibits human immunodeficiency virus type 1 transfer to CD4+ T cells. Antimicrob Agents Chemother.

[44] Peterson JA, Hamosh M, Scallan CD, Ceriani RL, Henderson TR, Mehta NR (1998). Milk fat globule glycoproteins in human milk and in gastric aspirates of mother's milk-fed preterm infants. Pediatr Res.

[45] Kvistgaard AS, Pallesen LT, Arias CF, López S, Petersen TE, Heegaard CW (2004). Inhibitory effects of human and bovine milk constituents on rotavirus infections. J Dairy Sci.

[46] Vestergaard EM, Nexo E, Wendt A, Guthmann F. (2008). Trefoil factors in human milk. Early Hum Dev.

[47] Barrera GJ, Sanchez G, Gonzalez JE. (2012). Trefoil factor 3 isolated from human breast milk downregulates cytokines (IL8 and IL6) and promotes human beta defensin (hBD2 and hBD4) expression in intestinal epithelial cells HT-29. Bosn J Basic Med Sci.

[48] Vidal K, van den Broek P, Lorget F, Donnet-Hughes A. (2004). Osteoprotegerin in human milk: a potential role in the regulation of bone metabolism and immune development. Pediatr Res.

[49] Zhao P, Zhang S, Liu L, Pang X, Yang Y, Lu J (2018). Differences in the triacylglycerol and fatty acid compositions of human colostrum and mature milk. J Agric Food Chem.

[50] Ganapathy S. (2009). Long chain polyunsaturated fatty acids and immunity in infants. Indian Pediatr.

[51] Wu J, Gong RL, Hu QF, Chen XT, Zhao W, Chen TX. (2019). Immunoregulatory effect of human β-defensin 1 on neonatal cord blood monocyte-derived dendritic cells and T cells. Mol Immunol.

[52] Baricelli J, Rocafull MA, Vázquez D, Bastidas B, Báez-Ramirez E, Thomas LE. (2015). β-defensin-2 in breast milk displays a broad antimicrobial activity against pathogenic bacteria. J Pediatr (Rio J).

[53] Li D, Chen P, Shi T, Mehmood A, Qiu J. (2021). HD5 and LL-37 Inhibit SARS-CoV and SARS-CoV-2 Binding to Human ACE2 by Molecular Simulation. Interdiscip Sci.

[54] Armogida SA, Yannaras NM, Melton AL, Srivastava MD. (2004). Identification and quantification of innate immune system mediators in human breast milk. Allergy Asthma Proc.

[55] Niyonsaba F, Iwabuchi K, Someya A, Hirata M, Matsuda H, Ogawa H (2002). A cathelicidin family of human antibacterial peptide LL-37 induces mast cell chemotaxis. Immunology.

[56] Hodgkinson A, Wall C, Wang W, Szeto IM, Ye W, Day L. (2021). Nucleotides: an updated review of their concentration in breast milk. Nutr Res.

[57] Xi Y, Jiang X, Li R, Chen M, Song W, Li X (2016). The levels of human milk microRNAs and their association with maternal weight characteristics. Eur J Clin Nutr.

[58] Carrillo-Lozano E, Sebastián-Valles F, Knott-Torcal C. (2020). Circulating microRNAs in breast milk and their potential impact on the infant. Nutrients.

[59] Liao Y, Alvarado R, Phinney B, Lönnerdal B. (2011). Proteomic characterization of human milk whey proteins during a twelve-month lactation period. J Proteome Res.

[60] Angelucci L, Patacchioli FR, Scaccianoce S, Di Sciullo A, Cardillo A, Maccari S. (1985). A model for later-life effects of perinatal drug exposure: maternal hormone mediation. Neurobehav Toxicol Teratol.

[61] Italianer MF, Naninck EFG, Roelants JA, van der Horst GTJ, Reiss IKM, Goudoever JBV (2020). Circadian variation in human milk composition, a systematic review. Nutrients.

[62] Vizzari G, Morniroli D, Ceroni F, Verduci E, Consales A, Colombo L (2021). Human milk, more than simple nourishment. Children.

[63] Bernt KM, Walker WA (1999). Human milk as a carrier of biochemical messages. Acta Paediatr Suppl.

[64] Fields DA, Demerath EW. (2012). Relationship of insulin, glucose, leptin, IL-6 and TNF-α in human breast milk with infant growth and body composition. Pediatr Obes.

[65] Savino F, Fissore MF, Liguori SA, Oggero R. (2009). Can hormones contained in mothers' milk account for the beneficial effect of breast-feeding on obesity in children?. Clin Endocrinol (Oxf).

[66] Palou A, Picó C. (2009). Leptin intake during lactation prevents obesity and affects food intake and food preferences in later life. Appetite.

[67] Aydin S, Ozkan Y, Kumru S. (2006). Ghrelin is present in human colostrum, transitional and mature milk. Peptides.

[68] Adkins Y, Lönnerdal B. (2003). Potential host-defense role of a human milk vitamin B-12-binding protein, haptocorrin, in the gastrointestinal tract of breastfed infants, as assessed with porcine haptocorrin in vitro. Am J Clin Nutr.

[69] York DJ, Smazal AL, Robinson DT, De Plaen IG. (2021). Human milk growth factors and their role in NEC prevention: a narrative review. Nutrients.

[70] Ozgurtas T, Aydin I, Turan O, Koc E, Hirfanoglu IM, Acikel CH (2010). Vascular endothelial growth factor, basic fibroblast growth factor, insulin-like growth factor-I and platelet-derived growth factor levels in human milk of mothers with term and preterm neonates. Cytokine.

[71] Lepage P, Van de Perre P. (2012). The immune system of breast milk: antimicrobial and anti-inflammatory properties. Adv Exp Med Biol.

[72] Kiełbasa A, Gadzała-Kopciuch R, Buszewski B. (2021). Cytokines-biogenesis and their role in human breast milk and determination. Int J Mol Sci.

[73] Pabst HF, Godel J, Grace M, Cho H, Spady DW. (1989). Effect of breast-feeding on immune response to BCG vaccination. Lancet.

[74] Hanson LA, Korotkova M, Telemo E. (2003). Breast-feeding, infant formulas, and the immune system. Ann Allergy Asthma Immunol.

[75] Penttila IA. (2010). Milk-derived transforming growth factor-beta and the infant immune response. J Pediatr.

[76] Kverka M, Burianova J, Lodinova-Zadnikova R, Kocourkova I, Cinova J, Tuckova L (2007). Cytokine profiling in human colostrum and milk by protein array. Clin Chem.

[77] Buescher ES, Malinowska I. (1996). Soluble receptors and cytokine antagonists in human milk. Pediatr Res.

[78] Agarwal S, Karmaus W, Davis S, Gangur V. (2011). Immune markers in breast milk and fetal and maternal body fluids: a systematic review of perinatal concentrations. J Hum Lact.

[79] Wolf M, Moser B. (2012). Antimicrobial activities of chemokines: not just a side-effect?. Front Immunol.

[80] Zhu J, Dingess KA, Mank M, Stahl B, Heck AJR. (2021). Personalized profiling reveals donor- and lactation-specific trends in the human milk proteome and peptidome. J Nutr.

[81] Brandtzaeg P. (2003). Mucosal immunity: integration between mother and the breast-fed infant. Vaccine.

[82] Zhou Q, Li M, Wang X, Li Q, Wang T, Zhu Q (2012). Immune-related microRNAs are abundant in breast milk exosomes. Int J Biol Sci.

[83] Alsaweed M, Hartmann PE, Geddes DT, Kakulas F. (2015). MicroRNAs in breastmilk and the lactating breast: potential immunoprotectors and developmental regulators for the infant and the mother. Int J Environ Res Public Health.

[84] Kim JH, Bode L, Ogra PL, Wilson CB, Nizet V, Maldonado YA, Remington JS, Klein JO (2016). Remington and Klein's infectious diseases of the fetus and newborn infant.

[85] Michie CA, Tantscher E, Schall T, Rot A. (1998). Physiological secretion of chemokines in human breast milk. Eur Cytokine Netw.

[86] Zhou L, Yoshimura Y, Huang Y, Suzuki R, Yokoyama M, Okabe M (2000). Two independent pathways of maternal cell transmission to offspring: through placenta during pregnancy and by breast-feeding after birth. Immunology.

[87] Jain L, Vidyasagar D, Xanthou M, Ghai V, Shimada S, Blend M. (1989). In vivo distribution of human milk leucocytes after ingestion by newborn baboons. Arch Dis Child.

[88] Duale A, Singh P, Al Khodor S (2021). Breast milk: a meal worth having. Front Nutr..

[89] Consales A, Cerasani J, Sorrentino G, Morniroli D, Colombo L, Mosca F (2022). The hidden universe of human milk microbiome: origin, composition, determinants, role, and future perspectives. Eur J Pediatr.

[90] Murphy K, Curley D, O'Callaghan TF, O'Shea CA, Dempsey EM, O'Toole PW (2017). The composition of human milk and infant faecal microbiota over the first three months of life: a pilot study. Sci Rep..

[91] Martín V, Maldonado-Barragán A, Moles L, Rodriguez-Baños M, Campo RD, Fernández L (2012). Sharing of bacterial strains between breast milk and infant feces. J Hum Lact.

[92] Jost T, Lacroix C, Braegger CP, Rochat F, Chassard C. (2014). Vertical mother-neonate transfer of maternal gut bacteria via breastfeeding. Environ Microbiol.

[93] Ruiz L, Bacigalupe R, García-Carral C, Boix-Amoros A, Argüello H, Silva CB (2019). Microbiota of human precolostrum and its potential role as a source of bacteria to the infant mouth. Sci Rep.

[94] Rescigno M, Urbano M, Valzasina B, Francolini M, Rotta G, Bonasio R (2001). Dendritic cells express tight junction proteins and penetrate gut epithelial monolayers to sample bacteria. Nat Immunol.

[95] Boix-Amorós A, Collado MC, Mira A (2016). Relationship between milk microbiota, bacterial load, macronutrients, and human cells during lactation. Front Microbiol.

[96] Perez PF, Doré J, Leclerc M, Levenez F, Benyacoub J, Serrant P (2007). Bacterial imprinting of the neonatal immune system: lessons from maternal cells?. Pediatrics.

[97] Latuga MS, Stuebe A, Seed PC (2014). A review of the source and function of microbiota in breast milk. Semin Reprod Med.

[98] Fernández L, Langa S, Martín V, Maldonado A, Jiménez E, Martín R (2013). The human milk microbiota: origin and potential roles in health and disease. Pharmacol Res.

[99] Vermeer C. (2012). Vitamin K: the effect on health beyond coagulation - an overview. Food Nutr Res.

[100] Goldman AS. (1993). The immune system of human milk: antimicrobial, antiinflammatory and immunomodulating properties. Pediatr Infect Dis J.

[101] Conley ME, Delacroix DL. (1987). Intravascular and mucosal immunoglobulin A: two separate but related systems of immune defense?. Ann Intern Med.

[102] Demers-Mathieu V, Huston RK, Markell AM, McCulley EA, Martin RL, Dallas DC. (2021). Impact of pertussis-specific IgA, IgM, and IgG antibodies in mother's own breast milk and donor breast milk during preterm infant digestion. Pediatr Res.

[103] Mathias A, Corthésy B. (2011). N-Glycans on secretory component: mediators of the interaction between secretory IgA and gram-positive commensals sustaining intestinal homeostasis. Gut Microbes.

[104] Ogra SS, Ogra PL. (1978). Immunologic aspects of human colostrum and milk. I. Distribution characteristics and concentrations of immunoglobulins at different times after the onset of lactation. J Pediatr.

[105] Victora CG, Bahl R, Barros AJ, França GV, Horton S, Krasevec J (2016). Breastfeeding in the 21st century: epidemiology, mechanisms, and lifelong effect. Lancet.

[106] Demers-Mathieu V, Underwood MA, Beverly RL, Nielsen SD, Dallas DC. (2018). Comparison of human milk immunoglobulin survival during gastric digestion between preterm and term infants. Nutrients.

[107] Carbonare SB, Silva ML, Palmeira P, Carneiro-Sampaio MM. (1997). Human colostrum IgA antibodies reacting to enteropathogenic Escherichia coli antigens and their persistence in the faeces of a breastfed infant. J Diarrhoeal Dis Res.

[108] Jasion VS, Burnett BP. (2015). Survival and digestibility of orally-administered immunoglobulin preparations containing IgG through the gastrointestinal tract in humans. Nutr J.

[109] Maertens K, De Schutter S, Braeckman T, Baerts L, Van Damme P, De Meester I (2014). Breastfeeding after maternal immunisation during pregnancy: providing immunological protection to the newborn: a review. Vaccine.

[110] Healy CM, Rench MA, Swaim LS, Timmins A, Vyas A, Sangi-Haghpeykar H (2020). Kinetics of maternal pertussis-specific antibodies in infants of mothers vaccinated with tetanus, diphtheria and acellular pertussis (Tdap) during pregnancy. Vaccine.

[111] Cherry JD. (2015). Tetanus-diphtheria-pertussis immunization in pregnant women and the prevention of pertussis in young infants. Clin Infect Dis.

[112] Shahid NS, Steinhoff MC, Roy E, Begum T, Thompson CM, Siber GR (2002). Placental and breast transfer of antibodies after maternal immunization with polysaccharide meningococcal vaccine: a randomized, controlled evaluation. Vaccine.

[113] Shahid NS, Steinhoff MC, Hoque SS, Begum T, Thompson C, Siber GR. (1995). Serum, breast milk, and infant antibody after maternal immunisation with pneumococcal vaccine. Lancet.

[114] Munoz FM, Englund JA, Cheesman CC, Maccato ML, Pinell PM, Nahm MH (2001). Maternal immunization with pneumococcal polysaccharide vaccine in the third trimester of gestation. Vaccine.

[115] Lehmann D, Pomat WS, Riley ID, Alpers MP (2003). Studies of maternal immunisation with pneumococcal polysaccharide vaccine in Papua New Guinea. Vaccine.

[116] Obaro SK, Deubzer HE, Newman VO, Adegbola RA, Greenwood BM, Henderson DC. (2004). Serotype-specific pneumococcal antibodies in breast milk of Gambian women immunized with a pneumococcal polysaccharide vaccine during pregnancy. Pediatr Infect Dis J.

[117] Deubzer HE, Obaro SK, Newman VO, Adegbola RA, Greenwood BM, Henderson DC. (2004). Colostrum obtained from women vaccinated with pneumococcal vaccine during pregnancy inhibits epithelial adhesion of Streptococcus pneumoniae. J Infect Dis.

[118] Schlaudecker EP, Steinhoff MC, Omer SB, McNeal MM, Roy E, Arifeen SE (2013). IgA and neutralizing antibodies to influenza a virus in human milk: a randomized trial of antenatal influenza immunization. PLoS One.

[119] Abu Raya B, Srugo I, Kessel A, Peterman M, Bader D, Peri R (2014). The induction of breast milk pertussis specific antibodies following gestational tetanus-diphtheria-acellular pertussis vaccination. Vaccine.

[120] Lima L, Molina MDGF, Pereira BS, Nadaf MLA, Nadaf MIV, Takano OA (2019). Acquisition of specific antibodies and their influence on cell-mediated immune response in neonatal cord blood after maternal pertussis vaccination during pregnancy. Vaccine.

[121] Orije MRP, Larivière Y, Herzog SA, Mahieu LM, Van Damme P, Leuridan E (2021). Breast milk antibody levels in Tdap-vaccinated women after preterm delivery. Clin Infect Dis.

[122] Brady RC, Jackson LA, Frey SE, Shane AL, Walter EB, Swamy GK (2018). Randomized trial comparing the safety and antibody responses to live attenuated versus inactivated influenza vaccine when administered to breastfeeding women. Vaccine.

[123] Oyedele OO, Odemuyiwa SO, Ammerlaan W, Muller CP, Adu FD (2005). Passive immunity to measles in the breastmilk and cord blood of some nigerian subjects. J Trop Pediatr.

[124] Bohlke K, Galil K, Jackson LA, Schmid DS, Starkovich P, Loparev VN (2003). Postpartum varicella vaccination: is the vaccine virus excreted in breast milk?. Obstet Gynecol.

[125] Alain S, Dommergues MA, Jacquard AC, Caulin E, Launay O. (2012). State of the art: Could nursing mothers be vaccinated with attenuated live virus vaccine?. Vaccine.

[126] Losonsky GA, Fishaut JM, Strussenberg J, Ogra PL. (1982). Effect of immunization against rubella on lactation products. I. Development and characterization of specific immunologic reactivity in breast milk. J Infect Dis.

[127] Losonsky GA, Fishaut JM, Strussenberg J, Ogra PL. (1982). Effect of immunization against rubella on lactation products. II. Maternal-neonatal interactions. J Infect Dis.

[128] Buimovici-Klein E, Hite RL, Byrne T, Cooper LZ. (1977). Isolation of rubella virus in milk after postpartum immunization. J Pediatr.

[129] Diseases NCfIaR (2011). General recommendations on immunization — recommendations of the Advisory Committee on Immunization Practices (ACIP). MMWR Recomm Rep.

[130] Traiber C, Coelho-Amaral P, Ritter VR, Winge A. (2011). Infant meningoencephalitis caused by yellow fever vaccine virus transmitted via breastmilk. J Pediatr (Rio J).

[131] Imbert P, Moulin F, Mornand P, Méchaï F, Rapp C. (2010). Should yellow fever vaccination be recommended during pregnancy or breastfeeding?. Med Trop.

[132] (2006).

[133] Safadi MAP, Spinardi J, Swerdlow D, Srivastava A. (2022 Jul). COVID-19 Disease and vaccination in pregnant and lactating women. Am J Reprod Immunol.

[134] Al-Kuraishy HM, Al-Gareeb AI, Atanu FO, El-Zamkan MA, Diab HM, Ahmed AS (2021). Maternal transmission of SARS-CoV-2: safety of breastfeeding in infants born to infected mothers. Front Pediatr.

[135] Ellington S, Strid P, Tong VT, Woodworth K, Galang RR, Zambrano LD (2020). Characteristics of women of reproductive age with laboratory-confirmed SARS-CoV-2 infection by pregnancy Status - United States, January 22-June 7, 2020. MMWR Morb Mortal Wkly Rep.

[136] Juan J, Gil MM, Rong Z, Zhang Y, Yang H, Poon LC. (2020). Effect of coronavirus disease 2019 (COVID-19) on maternal, perinatal and neonatal outcome: systematic review. Ultrasound Obstet Gynecol.

[137] Papapanou M, Papaioannou M, Petta A, Routsi E, Farmaki M, Vlahos N (2021). Maternal and neonatal characteristics and outcomes of COVID-19 in pregnancy: an overview of systematic reviews. Int J Environ Res Public Health.

[138] Angelidou A, Sullivan K, Melvin PR, Shui JE, Goldfarb IT, Bartolome R (2021). Association of maternal perinatal SARS-CoV-2 infection with neonatal outcomes during the COVID-19 pandemic in Massachusetts. JAMA Netw Open.

[139] Fox A, Marino J, Amanat F, Krammer F, Hahn-Holbrook J, Zolla-Pazner S (2020). Robust and specific secretory IgA against SARS-CoV-2 detected in human milk. iScience.

[140] Pace RM, Williams JE, Järvinen KM, Belfort MB, Pace CDW, Lackey KA (2021). Characterization of SARS-CoV-2 RNA, antibodies, and neutralizing capacity in milk produced by women with COVID-19. mBio.

[141] Dong Y, Chi X, Hai H, Sun L, Zhang M, Xie WF (2020). Antibodies in the breast milk of a maternal woman with COVID-19. Emerg Microbes Infect.

[142] Preßler J, Fill Malfertheiner S, Kabesch M, Buntrock-Döpke H, Häusler S, Ambrosch A (2020). Postnatal SARS-CoV-2 infection and immunological reaction: a prospective family cohort study. Pediatr Allergy Immunol.

[143] Lebrão CW, Cruz MN, Silva MHD, Dutra LV, Cristiani C, Affonso Fonseca FL (2020). Early identification of IgA Anti-SARSCoV-2 in milk of mother with COVID-19 infection. J Hum Lact.

[144] Chambers C, Krogstad P, Bertrand K, Contreras D, Tobin NH, Bode L (2020). Evaluation for SARS-CoV-2 in breast milk from 18 infected women. JAMA.

[145] Mazur NI, Horsley NM, Englund JA, Nederend M, Magaret A, Kumar A (2019). Breast milk prefusion F immunoglobulin G as a correlate of protection against respiratory syncytial virus acute respiratory illness. J Infect Dis.

[146] Gray KJ, Bordt EA, Atyeo C, Deriso E, Akinwunmi B, Young N (2021). Coronavirus disease 2019 vaccine response in pregnant and lactating women: a cohort study. Am J Obstet Gynecol.

[147] Hahn-Zoric M, Carlsson B, Björkander J, Mellander L, Friman V, Padyukov L (1997). Variable increases of IgG and IgM antibodies in milk of IgA deficient women. Pediatr Allergy Immunol.

[148] Palmeira P, Costa-Carvalho BT, Arslanian C, Pontes GN, Nagao AT, Carneiro-Sampaio MM. (2009). Transfer of antibodies across the placenta and in breast milk from mothers on intravenous immunoglobulin. Pediatr Allergy Immunol.

[149] Quinello C, Quintilio W, Carneiro-Sampaio M, Palmeira P. (2010). Passive acquisition of protective antibodies reactive with Bordetella pertussis in newborns via placental transfer and breast-feeding. Scand J Immunol.

[150] Spatz DL, Davanzo R, Müller JA, Powell R, Rigourd V, Yates A (2020). Promoting and protecting human milk and breastfeeding in a COVID-19 world. Front Pediatr.

[151] Narayanaswamy V, Pentecost BT, Schoen CN, Alfandari D, Schneider SS, Baker R (2022). Neutralizing antibodies and cytokines in breast milk after coronavirus disease 2019 (COVID-19) mRNA vaccination. Obstet Gynecol.

[152] Yeo KT, Chia WN, Tan CW, Ong C, Yeo JG, Zhang J (2021). Neutralizing activity and SARS-CoV-2 vaccine mRNA persistence in serum and breastmilk after BNT162b2 vaccination in lactating women. Front Immunol.

[153] Perez SE, Luna Centeno LD, Cheng WA, Marentes Ruiz CJ, Lee Y, Congrave-Wilson Z (2022). Human milk SARS-CoV-2 antibodies up to 6 months after vaccination. Pediatrics.

[154] Calil VMLT, Palmeira P, Zheng Y, Krebs VLJ, Carvalho WB, Carneiro-Sampaio M. (2021). CoronaVac can induce the production of anti-SARS-CoV-2 IgA antibodies in human milk. Clinics (Sao Paulo).

[155] Valcarce V, Stafford LS, Neu J, Cacho N, Parker L, Mueller M (2021). Detection of SARS-CoV-2-specific IgA in the human milk of COVID-19 vaccinated lactating health care workers. Breastfeed Med.

[156] Lechosa-Muñiz C, Paz-Zulueta M, Mendez-Legaza JM, Irure-Ventura J, Cuesta González R, Calvo Montes J (2021). Induction of SARS-CoV-2-specific IgG and IgA in serum and milk with different SARS-CoV-2 vaccines in breastfeeding women: a cross-sectional study in Northern Spain. Int J Environ Res Public Health.

[157] Juncker HG, Mulleners SJ, van Gils MJ, de Groot CJM, Pajkrt D, Korosi A (2021). The levels of SARS-CoV-2 specific antibodies in human milk following vaccination. J Hum Lact.

[158] Baird JK, Jensen SM, Urba WJ, Fox BA, Baird JR. (2021). SARS-CoV-2 antibodies detected in mother's milk post-vaccination. J Hum Lact.

[159] Selma-Royo M, Bäuerl C, Mena-Tudela D, Aguilar-Camprubí L, Pérez-Cano FJ, Parra-Llorca A (2021). Anti-Sars-Cov-2 IgA and IgG in human milk after vaccination is dependent on vaccine type and previous sars-cov-2 exposure: a longitudinal study. MedRxiv.

[160] Perl SH, Uzan-Yulzari A, Klainer H, Asiskovich L, Youngster M, Rinott E (2021). SARS-CoV-2-specific antibodies in breast milk after COVID-19 vaccination of breastfeeding women. JAMA.

[161] Sadiq S, Arslan F. (2021). https://doi:10.1515/almed-2021-0077.

[162] Low JM, Gu Y, Ng MSF, Amin Z, Lee LY, Ng YPM (2021). Codominant IgG and IgA expression with minimal vaccine mRNA in milk of BNT162b2 vaccinees. NPJ Vaccines.

[163] Fouda GG, Yates NL, Pollara J, Shen X, Overman GR, Mahlokozera T (2011). HIV-specific functional antibody responses in breast milk mirror those in plasma and are primarily mediated by IgG antibodies. J Virol.

[164] Demers-Mathieu V, Mathijssen G, Dapra C, Do DM, Medo E. (2021). Active free secretory component and secretory IgA in human milk: do maternal vaccination, allergy, infection, mode of delivery, nutrition and active lifestyle change their concentrations?. Pediatr Res.

[165] Semenov DV, Kanyshkova TG, Karotaeva NA, Krasnorutskii MA, Kuznetsova IA, Buneva VN (2004). Catalytic nucleotide-hydrolyzing antibodies in milk and serum of clinically healthy human mothers. Med Sci Monit.

[166] Van de Perre P, Simonon A, Hitimana DG, Dabis F, Msellati P, Mukamabano B (1993). Infective and anti-infective properties of breastmilk from HIV-1-infected women. Lancet.

